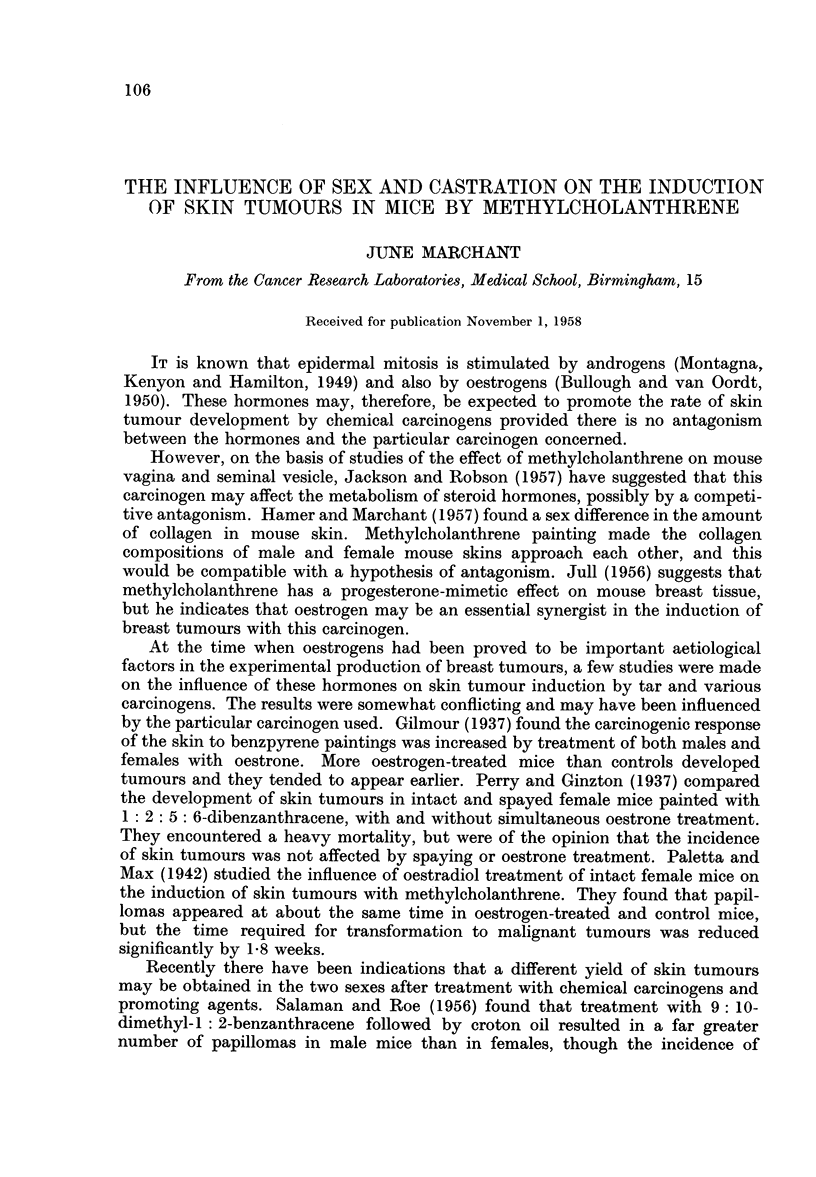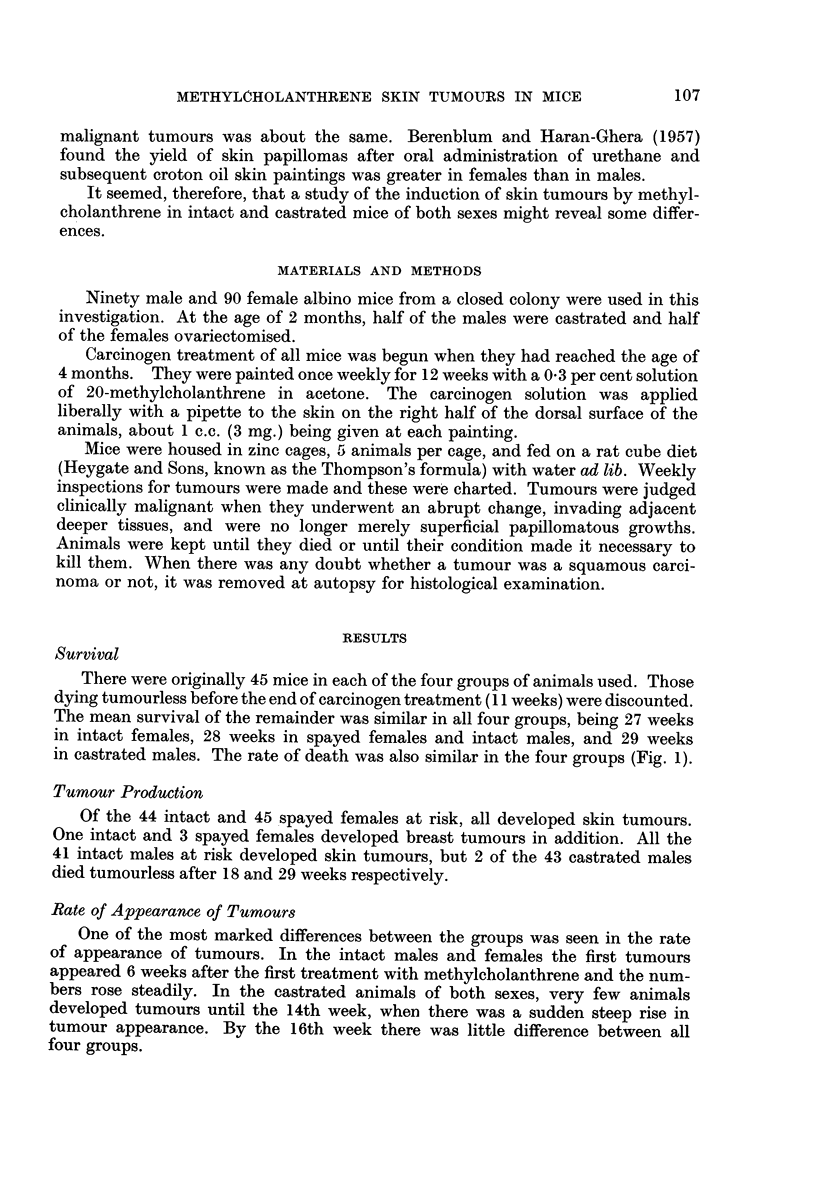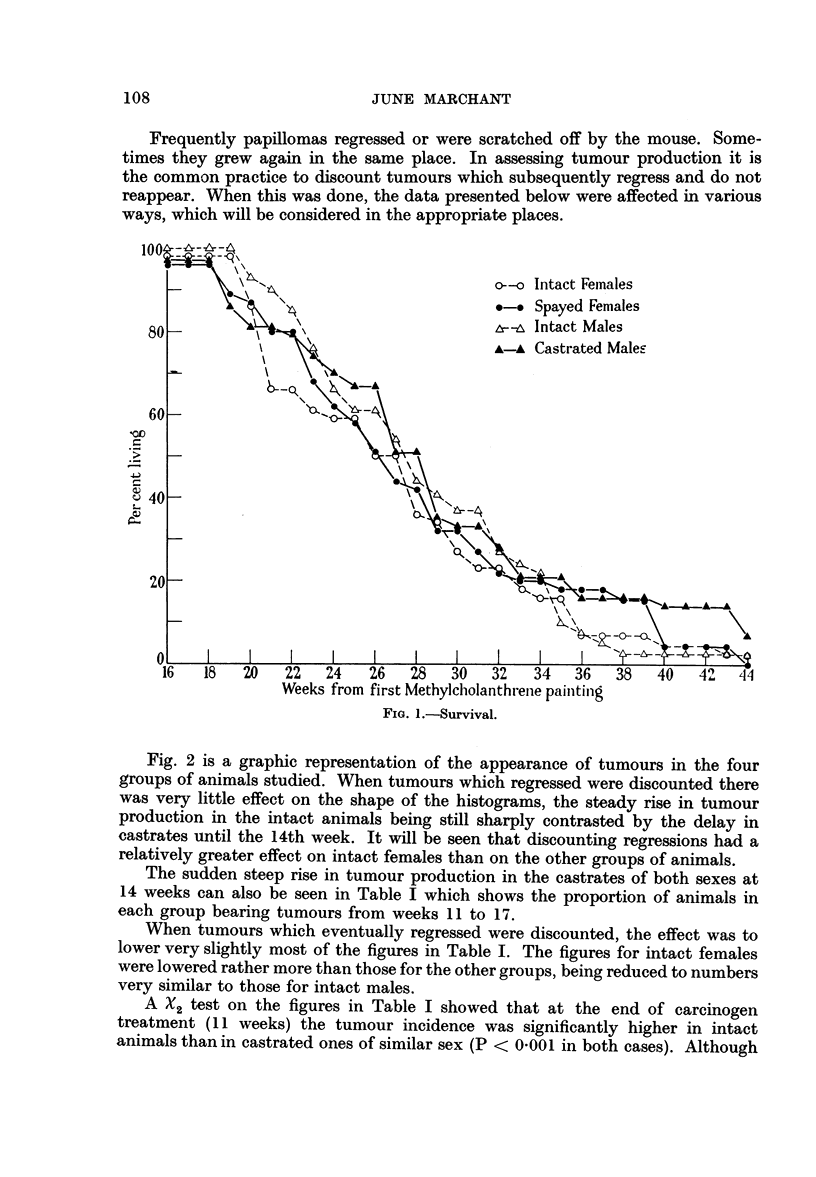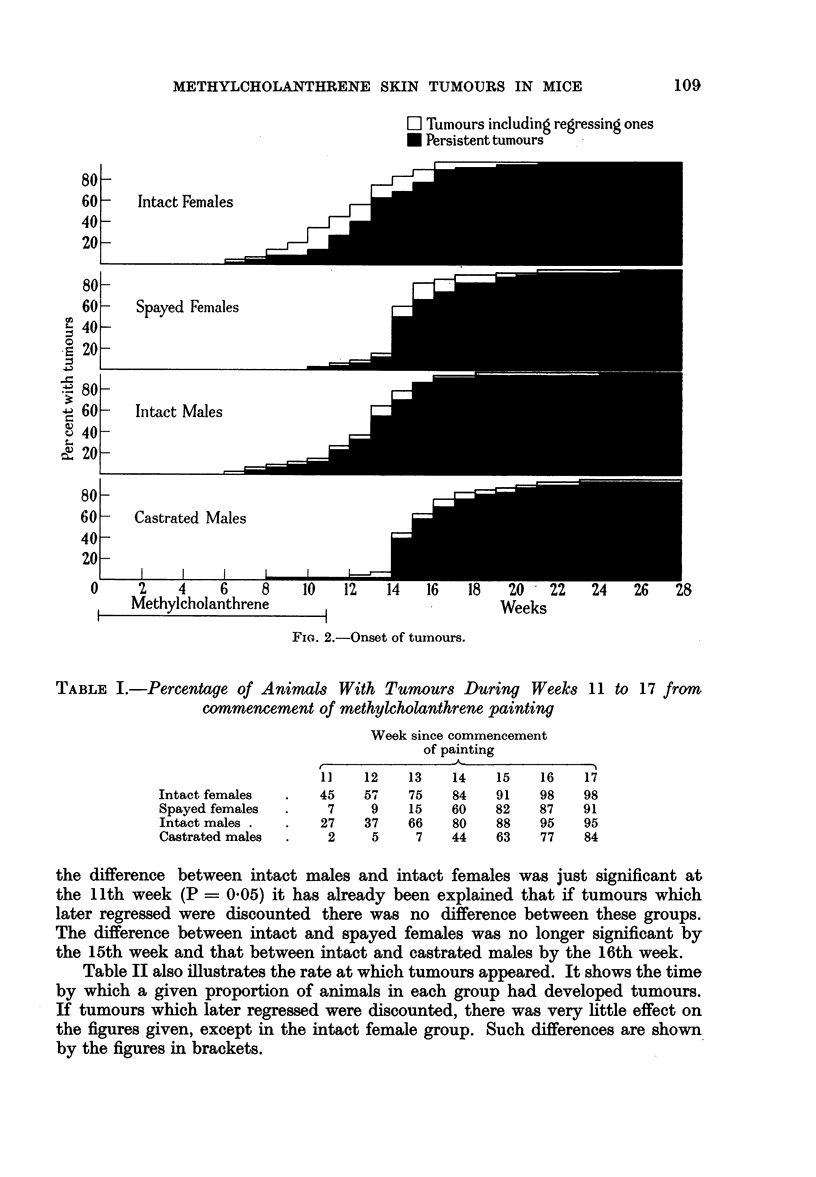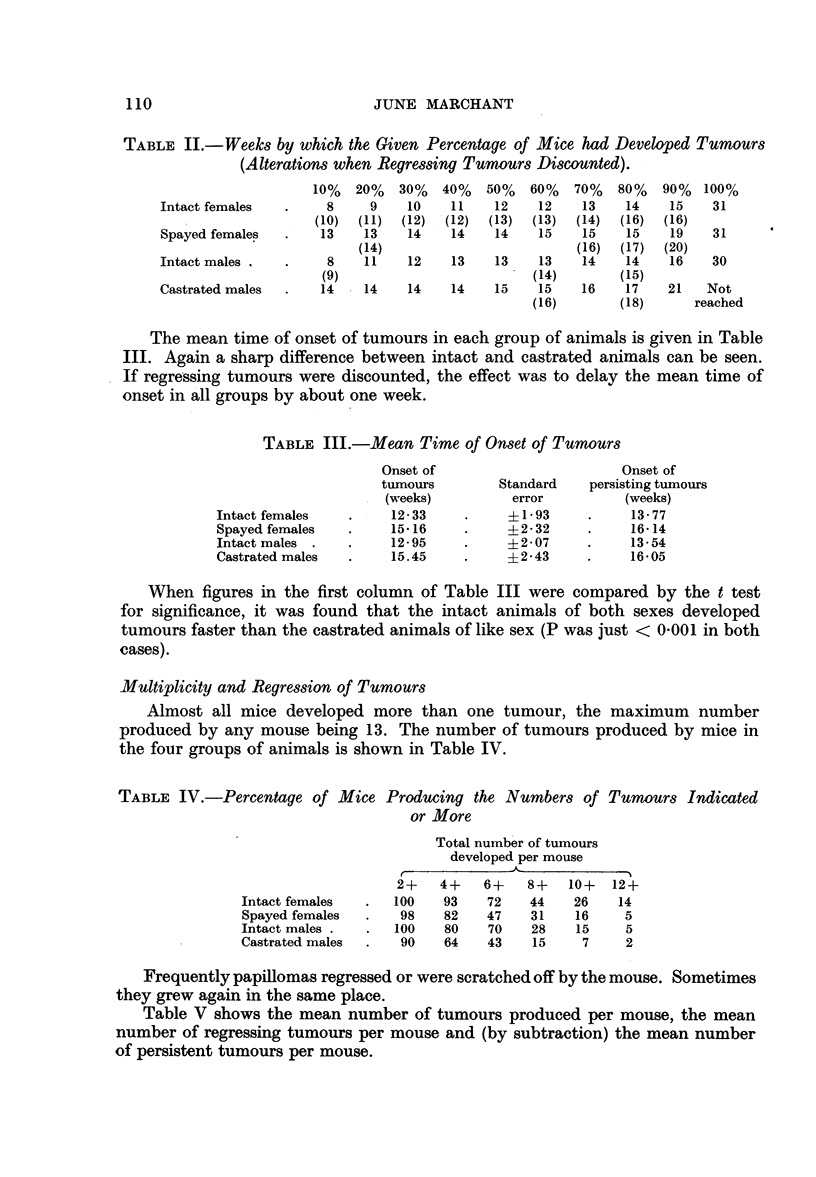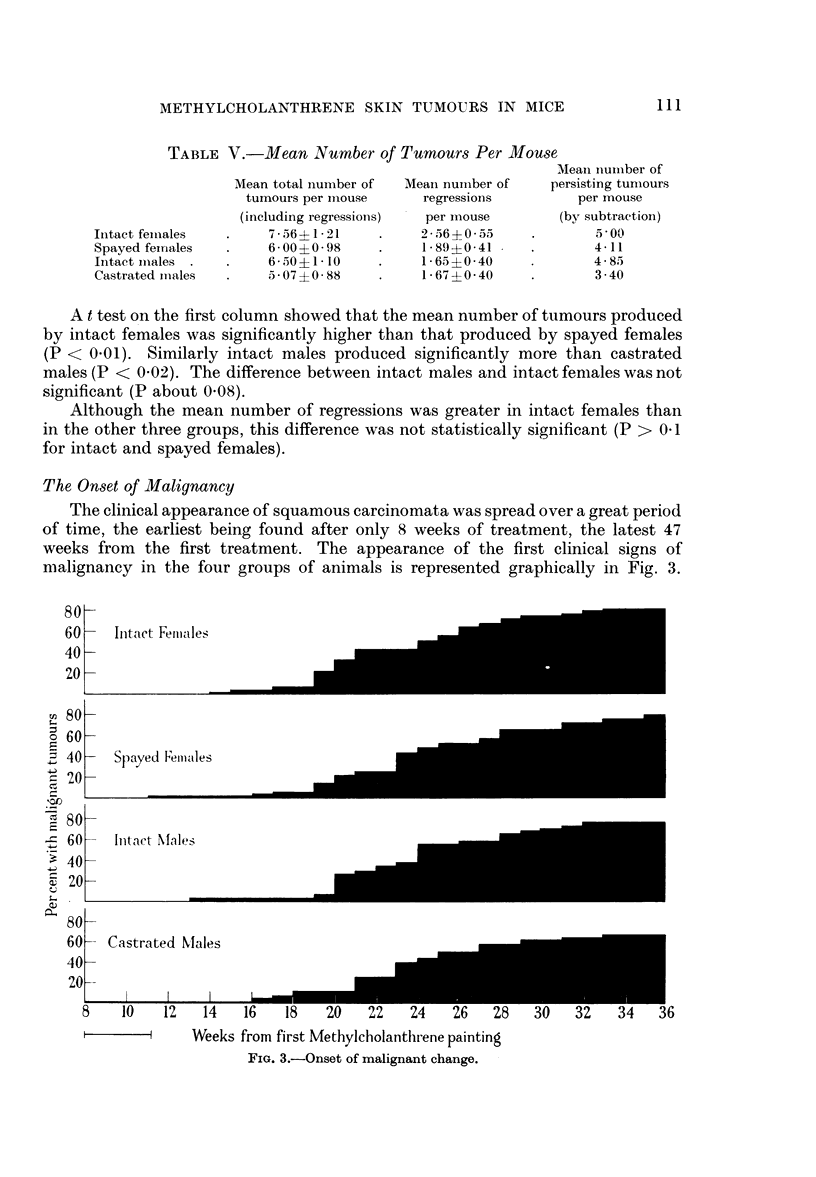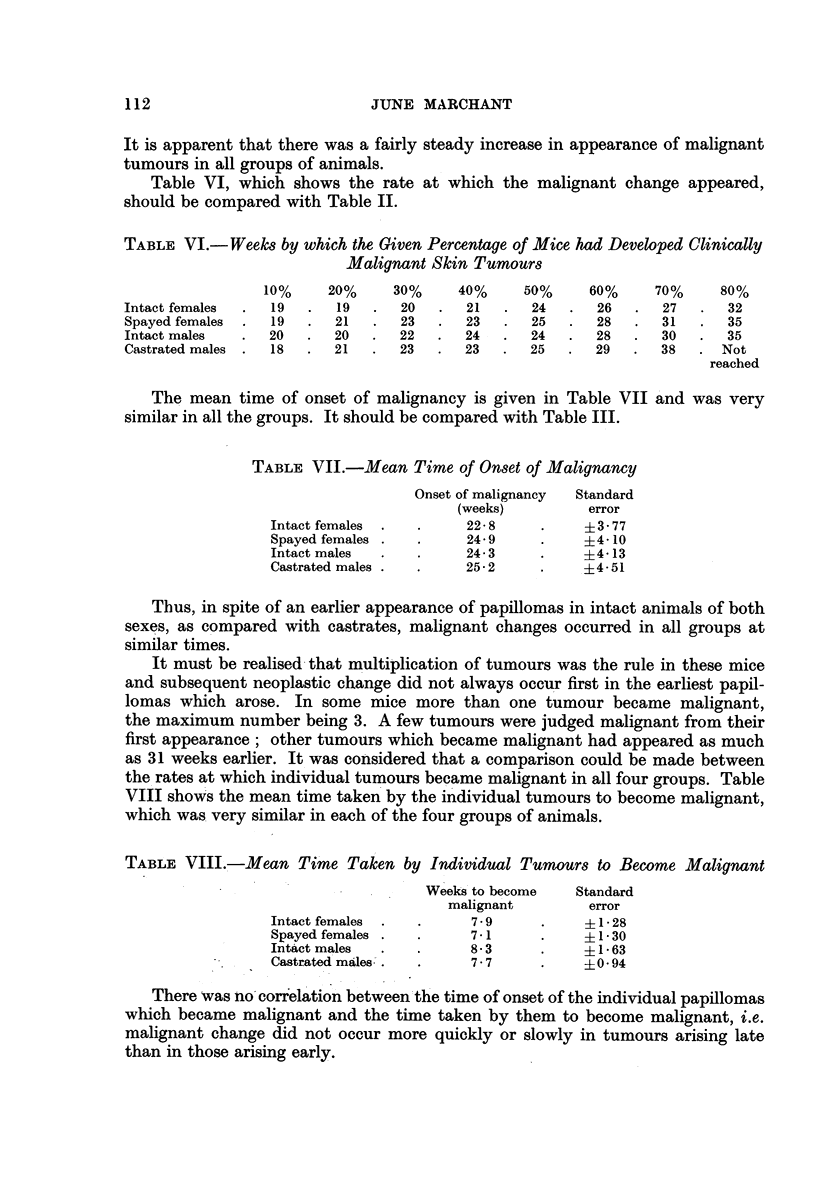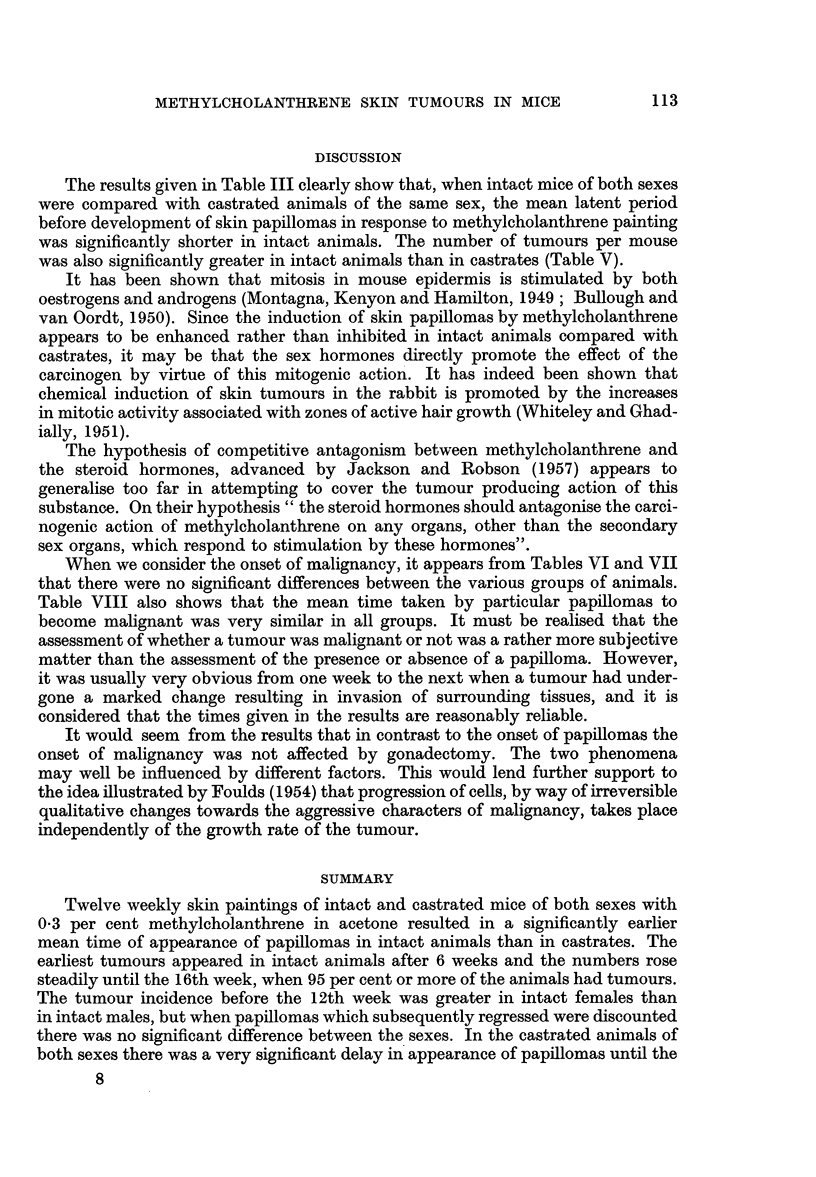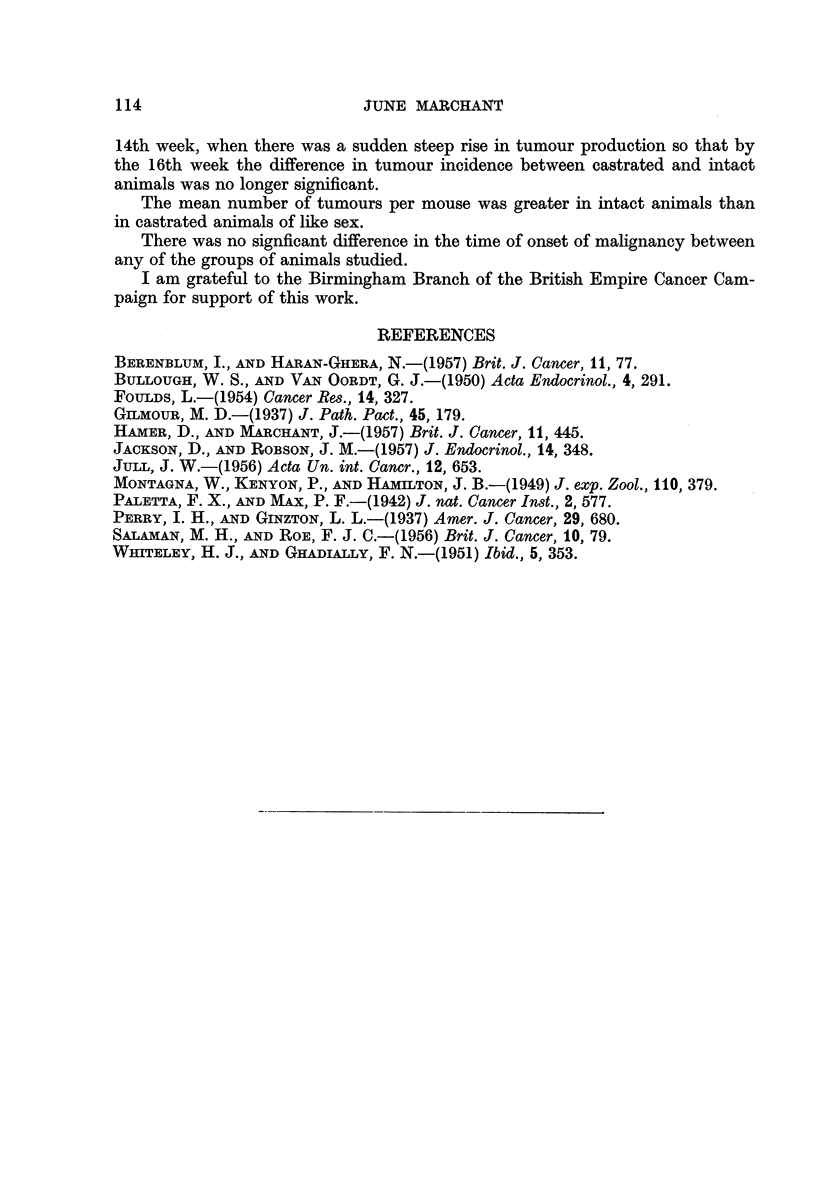# The Influence of Sex and Castration on the Induction of Skin Tumours in Mice by Methylcholanthrene

**DOI:** 10.1038/bjc.1959.15

**Published:** 1959-03

**Authors:** June Marchant


### THE INFLUENCE OF SEX AND CASTRATION ON THE INDUCTION OF SKIN TUMOURS IN MICE BY METHYLCHOLANTHRENE

JUNE MARCHANT

*From the Cancer Research Laboratories, Medical School, Birmingham,* 15

Received for publication November 1, 1958

It is known that epidermal mitosis is stimulated by androgens (Montagna, Kenyon and Hamilton, 1949) and also by oestrogens (Bullough and van Oordt, 1950). These hormones may, therefore, be expected to promote the rate of skin tumour development by chemical carcinogens provided there is no antagonism between the hormones and the particular carcinogen concerned.

However, on the basis of studies of the effect of methylcholanthrene on mouse vagina and seminal vesicle, Jackson and Robson (1957) have suggested that this carcinogen may affect the metabolism of steroid hormones, possibly by a competitive antagonism. Hamer and Marchant (1957) found a sex difference in the amount of collagen in mouse skin. Methylcholanthrene painting made the collagen compositions of male and female mouse skins approach each other, and this would be compatible with a hypothesis of antagonism. Jull (1956) suggests that methylcholanthrene has a progesterone-mimetic effect on mouse breast tissue, but he indicates that oestrogen may be an essential synergist in the induction of breast tumours with this carcinogen.

At the time when oestrogens had been proved to be important aetiological factors in the experimental production of breast tumours, a few studies were made on the influence of these hormones on skin tumour induction by tar and various carcinogens. The results were somewhat conflicting and may have been influenced by the particular carcinogen used. Gilmour (1937) found the carcinogenic response of the skin to benzpyrene paintings was increased by treatment of both males and females with oestrone. More oestrogen-treated mice than controls developed tumours and they tended to appear earlier. Perry and Ginzton (1937) compared the development of skin tumours in intact and spayed female mice painted with 1 : 2 : 5 : 6-dibenzanthracene, with and without simultaneous oestrone treatment. They encountered a heavy mortality, but were of the opinion that the incidence of skin tumours was not affected by spaying or oestrone treatment. Paletta and Max (1942) studied the influence of oestradiol treatment of intact female mice on the induction of skin tumours with methylcholanthrene. They found that papillomas appeared at about the same time in oestrogen-treated and control mice, but the time required for transformation to malignant tumours was reduced significantly by 1·8 weeks.

Recently there have been indications that a different yield of skin tumours may be obtained in the two sexes after treatment with chemical carcinogens and promoting agents. Salaman and Roe (1956) found that treatment with 9 : 10-dimethyl-1 : 2-benzanthracene followed by croton oil resulted in a far greater number of papillomas in male mice than in females, though the incidence of malignant tumours was about the same. Berenblum and Haran-Ghera (1957) found the yield of skin papillomas after oral administration of urethane and subsequent croton oil skin paintings was greater in females than in males.

It seemed, therefore, that a study of the induction of skin tumours by methylcholanthrene in intact and castrated mice of both sexes might reveal some differences.

### MATERIALS AND METHODS

Ninety male and 90 female albino mice from a closed colony were used in this investigation. At the age of 2 months, half of the males were castrated and half of the females ovariectomised.

Carcinogen treatment of all mice was begun when they had reached the age of 4 months. They were painted once weekly for 12 weeks with a 0·3 per cent solution of 20-methylcholanthrene in acetone. The carcinogen solution was applied liberally with a pipette to the skin on the right half of the dorsal surface of the animals, about 1 c.c. (3 mg.) being given at each painting.

Mice were housed in zinc cages, 5 animals per cage, and fed on a rat cube diet (Heygate and Sons, known as the Thompson's formula) with water *ad lib.* Weekly inspections for tumours were made and these were charted. Tumours were judged clinically malignant when they underwent an abrupt change, invading adjacent deeper tissues, and were no longer merely superficial papillomatous growths. Animals were kept until they died or until their condition made it necessary to kill them. When there was any doubt whether a tumour was a squamous carcinoma or not, it was removed at autopsy for histological examination.

### RESULTS

### *Survival*

There were originally 45 mice in each of the four groups of animals used. Those dying tumourless before the end of carcinogen treatment (11 weeks) were discounted. The mean survival of the remainder was similar in all four groups, being 27 weeks in intact females, 28 weeks in spayed females and intact males, and 29 weeks in castrated males. The rate of death was also similar in the four groups (Fig. 1).

### *Tumour Production*

Of the 44 intact and 45 spayed females at risk, all developed skin tumours. One intact and 3 spayed females developed breast tumours in addition. All the 41 intact males at risk developed skin tumours, but 2 of the 43 castrated males died tumourless after 18 and 29 weeks respectively.

### *Rate of Appearance of Tumours*

One of the most marked differences between the groups was seen in the rate of appearance of tumours. In the intact males and females the first tumours appeared 6 weeks after the first treatment with methylcholanthrene and the numbers rose steadily. In the castrated animals of both sexes, very few animals developed tumours until the 14th week, when there was a sudden steep rise in tumour appearance. By the 16th week there was little difference between all four groups.

Frequently papillomas regressed or were scratched off by the mouse. Sometimes they grew again in the same place. In assessing tumour production it is the common practice to discount tumours which subsequently regress and do not reappear. When this was done, the data presented below were affected in various ways, which will be considered in the appropriate places.

FIG. 1.—Survival.

Fig. 2 is a graphic representation of the appearance of tumours in the four groups of animals studied. When tumours which regressed were discounted there was very little effect on the shape of the histograms, the steady rise in tumour production in the intact animals being still sharply contrasted by the delay in castrates until the 14th week. It will be seen that discounting regressions had a relatively greater effect on intact females than on the other groups of animals.

The sudden steep rise in tumour production in the castrates of both sexes at 14 weeks can also be seen in Table I which shows the proportion of animals in each group bearing tumours from weeks 11 to 17.

When tumours which eventually regressed were discounted, the effect was to lower very slightly most of the figures in Table I. The figures for intact females were lowered rather more than those for the other groups, being reduced to numbers very similar to those for intact males.

A $X_2$ test on the figures in Table I showed that at the end of carcinogen treatment (11 weeks) the tumour incidence was significantly higher in intact animals than in castrated ones of similar sex ($P < 0{\cdot}001$ in both cases). Although

FIG. 2.—Onset of tumours.

TABLE I.—*Percentage of Animals With Tumours During Weeks* 11 *to* 17 *from commencement of methylcholanthrene painting*

| | Week since commencement of painting | | | | | | |
|---|---|---|---|---|---|---|---|
| | 11 | 12 | 13 | 14 | 15 | 16 | 17 |
| Intact females | 45 | 57 | 75 | 84 | 91 | 98 | 98 |
| Spayed females | 7 | 9 | 15 | 60 | 82 | 87 | 91 |
| Intact males | 27 | 37 | 66 | 80 | 88 | 95 | 95 |
| Castrated males | 2 | 5 | 7 | 44 | 63 | 77 | 84 |

the difference between intact males and intact females was just significant at the 11th week (P = 0·05) it has already been explained that if tumours which later regressed were discounted there was no difference between these groups. The difference between intact and spayed females was no longer significant by the 15th week and that between intact and castrated males by the 16th week.

Table II also illustrates the rate at which tumours appeared. It shows the time by which a given proportion of animals in each group had developed tumours. If tumours which later regressed were discounted, there was very little effect on the figures given, except in the intact female group. Such differences are shown by the figures in brackets.

TABLE II.—*Weeks by which the Given Percentage of Mice had Developed Tumours (Alterations when Regressing Tumours Discounted).*

| | 10% | 20% | 30% | 40% | 50% | 60% | 70% | 80% | 90% | 100% |
|---|---|---|---|---|---|---|---|---|---|---|
| Intact females | 8 (10) | 9 (11) | 10 (12) | 11 (12) | 12 (13) | 12 (13) | 13 (14) | 14 (16) | 15 (16) | 31 |
| Spayed females | 13 | 13 (14) | 14 | 14 | 14 | 15 | 15 (16) | 15 (17) | 19 (20) | 31 |
| Intact males | 8 (9) | 11 | 12 | 13 | 13 | 13 (14) | 14 | 14 (15) | 16 | 30 |
| Castrated males | 14 | 14 | 14 | 14 | 15 | 15 (16) | 16 | 17 (18) | 21 | Not reached |

The mean time of onset of tumours in each group of animals is given in Table III. Again a sharp difference between intact and castrated animals can be seen. If regressing tumours were discounted, the effect was to delay the mean time of onset in all groups by about one week.

TABLE III.—*Mean Time of Onset of Tumours*

| | Onset of tumours (weeks) | Standard error | Onset of persisting tumours (weeks) |
|---|---|---|---|
| Intact females | 12·33 | ±1·93 | 13·77 |
| Spayed females | 15·16 | ±2·32 | 16·14 |
| Intact males | 12·95 | ±2·07 | 13·54 |
| Castrated males | 15.45 | ±2·43 | 16·05 |

When figures in the first column of Table III were compared by the *t* test for significance, it was found that the intact animals of both sexes developed tumours faster than the castrated animals of like sex (P was just $< 0{\cdot}001$ in both cases).

*Multiplicity and Regression of Tumours*

Almost all mice developed more than one tumour, the maximum number produced by any mouse being 13. The number of tumours produced by mice in the four groups of animals is shown in Table IV.

TABLE IV.—*Percentage of Mice Producing the Numbers of Tumours Indicated or More*

| | Total number of tumours developed per mouse | | | | | |
|---|---|---|---|---|---|---|
| | 2+ | 4+ | 6+ | 8+ | 10+ | 12+ |
| Intact females | 100 | 93 | 72 | 44 | 26 | 14 |
| Spayed females | 98 | 82 | 47 | 31 | 16 | 5 |
| Intact males | 100 | 80 | 70 | 28 | 15 | 5 |
| Castrated males | 90 | 64 | 43 | 15 | 7 | 2 |

Frequently papillomas regressed or were scratched off by the mouse. Sometimes they grew again in the same place.

Table V shows the mean number of tumours produced per mouse, the mean number of regressing tumours per mouse and (by subtraction) the mean number of persistent tumours per mouse.

TABLE V.—*Mean Number of Tumours Per Mouse*

| | Mean total number of tumours per mouse (including regressions) | Mean number of regressions per mouse | Mean number of persisting tumours per mouse (by subtraction) |
|---|---|---|---|
| Intact females | 7·56±1·21 | 2·56±0·55 | 5·00 |
| Spayed females | 6·00±0·98 | 1·89±0·41 | 4·11 |
| Intact males | 6·50±1·10 | 1·65±0·40 | 4·85 |
| Castrated males | 5·07±0·88 | 1·67±0·40 | 3·40 |

A *t* test on the first column showed that the mean number of tumours produced by intact females was significantly higher than that produced by spayed females (P < 0·01). Similarly intact males produced significantly more than castrated males (P < 0·02). The difference between intact males and intact females was not significant (P about 0·08).

Although the mean number of regressions was greater in intact females than in the other three groups, this difference was not statistically significant (P > 0·1 for intact and spayed females).

*The Onset of Malignancy*

The clinical appearance of squamous carcinomata was spread over a great period of time, the earliest being found after only 8 weeks of treatment, the latest 47 weeks from the first treatment. The appearance of the first clinical signs of malignancy in the four groups of animals is represented graphically in Fig. 3.

FIG. 3.—Onset of malignant change.

It is apparent that there was a fairly steady increase in appearance of malignant tumours in all groups of animals.

Table VI, which shows the rate at which the malignant change appeared, should be compared with Table II.

TABLE VI.—*Weeks by which the Given Percentage of Mice had Developed Clinically Malignant Skin Tumours*

| | 10% | 20% | 30% | 40% | 50% | 60% | 70% | 80% |
|---|---|---|---|---|---|---|---|---|
| Intact females | 19 | 19 | 20 | 21 | 24 | 26 | 27 | 32 |
| Spayed females | 19 | 21 | 23 | 23 | 25 | 28 | 31 | 35 |
| Intact males | 20 | 20 | 22 | 24 | 24 | 28 | 30 | 35 |
| Castrated males | 18 | 21 | 23 | 23 | 25 | 29 | 38 | Not reached |

The mean time of onset of malignancy is given in Table VII and was very similar in all the groups. It should be compared with Table III.

TABLE VII.—*Mean Time of Onset of Malignancy*

| | Onset of malignancy (weeks) | Standard error |
|---|---|---|
| Intact females | 22·8 | ±3·77 |
| Spayed females | 24·9 | ±4·10 |
| Intact males | 24·3 | ±4·13 |
| Castrated males | 25·2 | ±4·51 |

Thus, in spite of an earlier appearance of papillomas in intact animals of both sexes, as compared with castrates, malignant changes occurred in all groups at similar times.

It must be realised that multiplication of tumours was the rule in these mice and subsequent neoplastic change did not always occur first in the earliest papillomas which arose. In some mice more than one tumour became malignant, the maximum number being 3. A few tumours were judged malignant from their first appearance; other tumours which became malignant had appeared as much as 31 weeks earlier. It was considered that a comparison could be made between the rates at which individual tumours became malignant in all four groups. Table VIII shows the mean time taken by the individual tumours to become malignant, which was very similar in each of the four groups of animals.

TABLE VIII.—*Mean Time Taken by Individual Tumours to Become Malignant*

| | Weeks to become malignant | Standard error |
|---|---|---|
| Intact females | 7·9 | ±1·28 |
| Spayed females | 7·1 | ±1·30 |
| Intact males | 8·3 | ±1·63 |
| Castrated males | 7·7 | ±0·94 |

There was no correlation between the time of onset of the individual papillomas which became malignant and the time taken by them to become malignant, *i.e.* malignant change did not occur more quickly or slowly in tumours arising late than in those arising early.

### DISCUSSION

The results given in Table III clearly show that, when intact mice of both sexes were compared with castrated animals of the same sex, the mean latent period before development of skin papillomas in response to methylcholanthrene painting was significantly shorter in intact animals. The number of tumours per mouse was also significantly greater in intact animals than in castrates (Table V).

It has been shown that mitosis in mouse epidermis is stimulated by both oestrogens and androgens (Montagna, Kenyon and Hamilton, 1949 ; Bullough and van Oordt, 1950). Since the induction of skin papillomas by methylcholanthrene appears to be enhanced rather than inhibited in intact animals compared with castrates, it may be that the sex hormones directly promote the effect of the carcinogen by virtue of this mitogenic action. It has indeed been shown that chemical induction of skin tumours in the rabbit is promoted by the increases in mitotic activity associated with zones of active hair growth (Whiteley and Ghadially, 1951).

The hypothesis of competitive antagonism between methylcholanthrene and the steroid hormones, advanced by Jackson and Robson (1957) appears to generalise too far in attempting to cover the tumour producing action of this substance. On their hypothesis " the steroid hormones should antagonise the carcinogenic action of methylcholanthrene on any organs, other than the secondary sex organs, which respond to stimulation by these hormones".

When we consider the onset of malignancy, it appears from Tables VI and VII that there were no significant differences between the various groups of animals. Table VIII also shows that the mean time taken by particular papillomas to become malignant was very similar in all groups. It must be realised that the assessment of whether a tumour was malignant or not was a rather more subjective matter than the assessment of the presence or absence of a papilloma. However, it was usually very obvious from one week to the next when a tumour had undergone a marked change resulting in invasion of surrounding tissues, and it is considered that the times given in the results are reasonably reliable.

It would seem from the results that in contrast to the onset of papillomas the onset of malignancy was not affected by gonadectomy. The two phenomena may well be influenced by different factors. This would lend further support to the idea illustrated by Foulds (1954) that progression of cells, by way of irreversible qualitative changes towards the aggressive characters of malignancy, takes place independently of the growth rate of the tumour.

### SUMMARY

Twelve weekly skin paintings of intact and castrated mice of both sexes with 0·3 per cent methylcholanthrene in acetone resulted in a significantly earlier mean time of appearance of papillomas in intact animals than in castrates. The earliest tumours appeared in intact animals after 6 weeks and the numbers rose steadily until the 16th week, when 95 per cent or more of the animals had tumours. The tumour incidence before the 12th week was greater in intact females than in intact males, but when papillomas which subsequently regressed were discounted there was no significant difference between the sexes. In the castrated animals of both sexes there was a very significant delay in appearance of papillomas until the

14th week, when there was a sudden steep rise in tumour production so that by the 16th week the difference in tumour incidence between castrated and intact animals was no longer significant.

The mean number of tumours per mouse was greater in intact animals than in castrated animals of like sex.

There was no signficant difference in the time of onset of malignancy between any of the groups of animals studied.

I am grateful to the Birmingham Branch of the British Empire Cancer Campaign for support of this work.

### REFERENCES

Berenblum, I., and Haran-Ghera, N.—(1957) *Brit. J. Cancer*, **11**, 77.
Bullough, W. S., and Van Oordt, G. J.—(1950) *Acta Endocrinol.*, **4**, 291.
Foulds, L.—(1954) *Cancer Res.*, **14**, 327.
Gilmour, M. D.—(1937) *J. Path. Pact.*, **45**, 179.
Hamer, D., and Marchant, J.—(1957) *Brit. J. Cancer*, **11**, 445.
Jackson, D., and Robson, J. M.—(1957) *J. Endocrinol.*, **14**, 348.
Jull, J. W.—(1956) *Acta Un. int. Cancr.*, **12**, 653.
Montagna, W., Kenyon, P., and Hamilton, J. B.—(1949) *J. exp. Zool.*, **110**, 379.
Paletta, F. X., and Max, P. F.—(1942) *J. nat. Cancer Inst.*, **2**, 577.
Perry, I. H., and Ginzton, L. L.—(1937) *Amer. J. Cancer*, **29**, 680.
Salaman, M. H., and Roe, F. J. C.—(1956) *Brit. J. Cancer*, **10**, 79.
Whiteley, H. J., and Ghadially, F. N.—(1951) *Ibid.*, **5**, 353.